# Hipersexuality in aripiprazole treatment : A case report

**DOI:** 10.1192/j.eurpsy.2022.1849

**Published:** 2022-09-01

**Authors:** M.V. López Rodrigo, M. Palomo Monge, P. Padilla Romero, A. Osca Oliver, M. Pérez Fominaya

**Affiliations:** 1 Hospital Nuestra Señora del Prado, Psiquiatría, Talavera de la Reina, Spain; 2 Hospital Nuestra Señora del Prado, Psiquiatria, Talavera de la Reina, Spain; 3 Hospital Universitario insular, Psiquiatría, Las Palmas de Gran Canaria, Spain

**Keywords:** hipersexuality, D2partialagonist

## Abstract

**Introduction:**

Aripiprazole is an antipsychotic that differs from the group in that it is a partial agonist of the D2 receptor, being also a partial agonist of 5HT1A and an antagonist of 5HT2A.Most antipsychotics cause a decrease in libido, affecting both sexual desire and function. According to the literature, D2 partial agonists can cause the appearance of compulsive behaviors as an adverse effect in 6-24% of patients. Among these behaviors you can find hypersexuality. In most cases, it subsides when treatment is stopped. We describe the case of a patient with bipolar disorder who develops hypersexual behaviors following the aripiprazole treatment. This is a 61-year-old bipolar patient receiving valproate and risperidone. It requires hospital admission due to manic symptoms where dysfunctional tremor is observed. Change from risperidone to aripiprazole. Subsequently, hypersexual behaviors appear, increased libido, obsession with sexual activities (compulsive masturbation with TV programs, mobile applications, cartoons) as well as delusional ideas about “receiving sexual gazes” with no other maniac symptomps.

**Objectives:**

To determine the possibility that hypersexuality was induced by treatment with aripiprazole.

**Methods:**

The appearance in the time line of hypersexuality after the change of treatment would be indicative of causality.

**Results:**

After switching back to risperidone, compulsive sexual behaviors disappear but not the delusional idea of being the focus of sexual gazes by everyone.

**Conclusions:**

Although it is not a common adverse effect, hypersexuality is listed in the literature as a rare adverse effect.

**Disclosure:**

No significant relationships.

